# “I don’t wanna die, but my brain insists that I should”: a big qualitative data approach to the lived experiences of suicidal thoughts

**DOI:** 10.3389/fpsyg.2024.1420287

**Published:** 2024-08-27

**Authors:** Lauro Estivalete Marchionatti, Rafael Ramos Amaral, Camila Barcellos, Samanta Duarte, André Cardoso Campello, Eduardo Virtuoso, Pedro Vieira da Silva Magalhães

**Affiliations:** ^1^Faculty of Medicine, Graduate Program in Psychiatry and Behavioral Sciences, Universidade Federal do Rio Grande do Sul, Porto Alegre, Brazil; ^2^Centro de Pesquisa Clínica, Hospital de Clínicas de Porto Alegre, Porto Alegre, Brazil; ^3^Instituto de Psiquiatria, Hospital das Clínicas, Faculdade de Medicina, Universidade de São Paulo, São Paulo, Brazil

**Keywords:** suicidal ideation, suicide, phenomenology, inductive, qualitative, lived experience

## Abstract

**Introduction:**

There remains a dearth of knowledge concerning the phenomenology of suicidal thoughts, with research focusing on reasons for feeling suicidal rather than their mental expression. While clinical interviews remain the standard phenomenological approach, such exploration of lived experiences may prove challenging for this sensitive topic. As a complementary alternative, the use of naturally-occurring online data is opportune for capturing elaborations on tabooed phenomena.

**Methods:**

In this phenomenological study, we present a thematic analysis on lived experiences of suicidal thoughts as spontaneously reported by non-identified users of a Reddit online board (r/Depression), collecting 668 posts using the search terms “suicidal ideation,” “suicidal thoughts,” and “suicide.” Codes were grouped into descriptive categories summarizing the properties of thoughts, their effects, and their relation to suicide. Then, an interpretative synthesis yielded global themes connecting salient meanings on the experience of suicidal thoughts.

**Results:**

With a long-term and recurring nature, thoughts of suicide appear in the form of vivid imagery and daydreaming’s, initially bringing relief to adverse feelings but eventually becoming conditioned and all-consuming. Rather than a wonderment, they are experienced as intrusive thoughts by people struggling to make meaning of their occurrence. When conciliating the presence of unwanted thoughts, users express intricate relations to wishing or not to die, as well as varying perceptions of control over actions or fear of suicidal behavior.

**Discussion:**

With an innovative application of big qualitative data into phenomenological analysis, this study contributes to an initial characterization of suicidal thoughts, uncovering findings that are not contemplated into current conceptualizations of suicidality. The analysis is limited by a restricted context of posts and unknown demographics, and further research with clinical interviews is warranted for in-depth exploration of suicidal thoughts.

## 1 Introduction

Suicidal thoughts are emotionally distressing and place a substantial burden on individuals (Khasakhala et al., 2011; Fischer et al., 2023). A complex phenomenon embedded within many determinants, suicidal thoughts may mean or represent different things and also express different forms of psychopathology (Khasakhala et al., 2011; White, 2016; Harmer et al., 2021). Nevertheless, contemporary suicidology has often approached it by inspecting associated factors, such as depression, loneliness, and low self-efficacy, attempting to uncover processes into ideation-to-action models of suicide (Van Orden et al., 2010; Tucker et al., 2018; Liu et al., 2020; Delgadillo et al., 2023). While significantly contributing to describing the emergence and progression of suicidal thoughts, relevant aspects may escape the conceptualizations of current models, particularly an in-depth individual account of experience and context. It has been argued that gaps remain in the phenomenology of suicidal thoughts, including attributes of their symptomatic expression such as function, course, and associated meanings (Oquendo and Baca-Garcia, 2014; White, 2016; House et al., 2020; Marchionatti and Magalhães, 2023).

Qualitative research can shed light on intricate phenomena by centering on lived experience as the focal point of inquiry (White, 2016; Fusar-Poli et al., 2022). Qualitative explorations of suicidal ideation have often revealed novel aspects not sufficiently captured by existing models of suicide, including the role of thoughts as a coping mechanism, the chronic and complex nature of its trajectory, or the association with suicidal imagery (Sturgeon and Morrissette, 2010; Denneson et al., 2020; Marchionatti and Magalhães, 2023). For instance, a thematic analysis of interviews with 50 veterans in the United States of America revealed that suicidal ideation is often chronic and complex, with fluctuations in frequency and intensity perceived as uncontrollable and unpredictable (Denneson et al., 2020). Similarly, narratives from Canadian adolescents led to the understanding of suicidal ideation as a way of coping with distress in the absence of a repertoire of strategies (Paulson and Everall, 2003). In a similar vein, a study with LGBTQIA+ men in New York recently diagnosed with HIV suggested that suicidality initiated a process that ultimately enhanced their sense of control over life, facilitating the acceptance of their diagnosis (Siegel and Meyer, 1999). Nevertheless, qualitative studies tend to focus on the contextual reasons for feeling suicidal and overcoming it, with considerably less emphasis on the very manifestation of suicidal thoughts (Harris et al., 2019; Castro-Ramirez et al., 2023; Søndergaard et al., 2023). For instance, interviews with Manitoban farmers primarily focused on factors triggering ideation, such as financial and health concerns common to the rural environment (Sturgeon and Morrissette, 2010). Conversely, adopting a phenomenological lens could contribute to approach suicidal thoughts as a mental object in itself, delineating possible expressions in the conscious mind (Fuchs et al., 2019; Messas et al., 2023; Summa, 2023).

Noteworthy, suicidal thoughts and ideation pose a challenging research inquiry as tabooed experiences are often concealed from close relationships, health professionals, and even therapists (Husky et al., 2016; McGillivray et al., 2022; Hallford et al., 2023). This enhances potential biases in traditional face-to-face interviews, particularly in terms of recruitment and social desirability reporting (Kristensen and Ravn, 2015; Bergen and Labonté, 2020; Höpfner and Promberger, 2023). An alternative lies in the use of big qual, an emerging method that incorporates large-scale datasets within a qualitative framework (Marchionatti et al., 2019). A main source of secondary data is the massive corpus of human expressions available on the internet, such as in forums, social media, blogs, and comments on webpages. Consisting of naturally occurring data, they offer a potential window into spontaneous elaborations that reflect fresh experiences, thereby minimizing influences from memory recall and research scripts (Robinson, 2022). Online bulletin boards are especially valuable for fostering textually rich discussions on particular subjects and enabling anonymous posting, potentially capturing perspectives that are underrepresented in traditional research (Maxwell et al., 2020; Osadchiy et al., 2020).

Reddit is an online bulletin platform that can provide data for mental health research, with the particular advantage of incentivizing non-identified posting on niched forums (Boettcher, 2021). As one of the largest online communities, Reddit has a diverse user base that includes people who may be at risk or who have been affected by suicide (Monselise and Yang, 2022). Through the platform’s user-generated content, researchers can gain insight into the attitudes, experiences, and risk factors associated with suicidality, offering a opportunity to study suicide-related communication (Mason et al., 2021; Slemon et al., 2021). Here, we report a thematic analysis of hundreds of user-generated posts on the platform where we examine the lived experience of suicidal thoughts adopting a phenomenological framework.

## 2 Materials and methods

We sought to analyze the expression of suicidal thoughts posted on Reddit, turning to this archive of secondary data for its volume of spontaneous, non-identified narratives on mental health topics. Big qualitative designs involve utilizing large datasets as a source of experiences that can be flexibly combined within qualitative traditions (Marchionatti et al., 2019). We offer a novel big data application within the realm of phenomenology, as we were interested in the subjective experience and sense-making of suicidal thoughts in the conscious mind. We employed thematic analysis as a method, drawing on previous theoretical orientation for conceptualizing it within phenomenological traditions (Sundler et al., 2019; Landrum and Davis, 2023). Our conceptual framework has a focus on lived experiences, where researchers suspend their assumptions to delve into participants’ firsthand accounts and meaning-making, constructing a detailed description and interpretation of psychological processes. We use a codebook approach for thematic analysis, integrating structured coding for a general account of findings and a reflexive synthesis that acknowledges researcher subjectivity as a valuable tool in the analytical process (Braun and Clarke, 2021, 2022).

Data was obtained from the social media Reddit, an internet board with over 430 million monthly users and 138 thousand active communities (Kastrenakes, 2020; Proferes et al., 2021). Reddit is structured around user-created forums known as “subreddits,” focused on specific topics, many of which relate to mental health-related issues. Individuals are registered under pseudonyms, making the platform particularly well-suited for researching sensitive topics, as the expectation of anonymity may increase trustworthiness and authentic reporting (Schrading et al., 2015; Proferes et al., 2021). We adopted the guidance provided by Caplan and Purser (2019) for undertaking qualitative inquiry using social media as an applicable framework.

Big quali approaches with secondary sources require the collection of research information from extensive datasets by identifying, retrieving, and processing pertinent data elements (Marchionatti et al., 2019; Mills, 2019). We drew on the “breadth-and-depth” method by Davidson et al. (2019), employing manual and automated procedures to assemble numerous pieces of information from a large archive yet retaining a richness of detail and nuance for qualitative interpretation. We initially screened several subreddits related to mental health for posts about suicidal thoughts (i.e., r/depression, r/suicidewatch, r/mentalhealth, r/bipolardisorder, r/suicidalthoughts). We decided to include only data from the subreddit r/depression to narrow the scope and increase the consistency of the analysis as it contained a more than sufficient number of posts. We developed a script in Python using the PRAW library and Jupyter Notebook that extracted the relevant posts in the subreddit (see Supplementary Table 1 for source code; Kluyver et al., 2016; Boe, 2020). The program interacted with the platform, searching for all posts (as well as their associated comments and replies) containing the terms “suicidal ideation,” “suicidal thoughts,” or “suicide,” and extracting their content into a text file. A total of 668 thread posts were initially obtained and inspected to exclude duplicates and irrelevant posts, resulting in 495 discussions, each containing at least one participant’s input. These were then uploaded to NVivo version 1.5.1 for managing the analysis (QSR International Pty Ltd, 2020). While there is no consensual criteria for big qual, our compiled dataset far exceeds suggested minimum thresholds, often on the order of 100 participants (Marchionatti et al., 2019).

We employed an inductive thematic analysis utilizing the codebook approach delineated by Braun and Clarke (2021, 2022). Our unit of analysis contained restricted text, usually a single or few paragraphs from thread posts and responses that collectively formed an extensive data corpus. Although sharing conceptual principles with interpretative phenomenological analysis, the nature of our data precluded the idiographic approach of in-depth analysis of single cases (Braun and Clarke, 2021). Thematic analysis was then chosen for its long tradition in phenomenological research and its adaptability to naturalistic datasets, suiting our endeavor of examining patterns of meaning in a collection of brief texts [refer to Robinson (2022) for a discussion]. Drawing on the tiered approach of Fletcher and StGeorge (2011) analysis of an online supportive chat room, we constructed a two-level procedure encompassing a descriptive summary and an interpretative stance. The descriptive process involved acquiring familiarity with the dataset through extensive reading and performing basic coding by identifying patterns of meaning in text extracts that were close to the participant’s own words; those were gathered into summarizing categories for a descriptive account of low-order findings across the dataset. At a higher-level interpretative stance, codes and categories were inspected for salient meanings and connections, generating unifying themes that synthesize findings through the researchers’ analytical lens. At this level, a cohesive narrative emerges to provide a theoretical model for the phenomenon, arising from the intersection of empirical data and the researchers’ interpretative insights. This two-level approach aligns with the flexibility embedded within codebook thematic analysis, which permits structured coding generating topic summaries that “map” a given phenomenon, as well as deriving reflective themes with metatheoretical perspectives anchored at codes and categories (Braun and Clarke, 2021, 2022).

These analytical steps were mainly conducted by one of the authors (LEM), being extensively discussed with other members of the team (CB, PVSM, and RR) in regular sessions to gain interpretative perspectives and enrich reflexivity. A fourth researcher (ACC) was engaged in the final stages of the analysis, operating as an external consultant blinded to the initial process for verifying whether analytical claims were substantiated by the data. All researchers self-identified as cisgender male or female. LEM is a psychiatrist and PhD candidate with at least 3 years of experience working with qualitative research; PM is a professor of psychiatry working with people with suicidal ideation and qualitative methods in several projects in the past 10 years; ACC is a psychiatrist with academic experience in phenomenology and social theory; RR is a medical resident in his first year of training in mental health practice; CB is an undergraduate medical student with no previous training in psychiatry or psychology. The authors reflect on their mental health practice/research background, acknowledging a potential influence on examining phenomena through the lens of psychopathology (for instance, the codification of suicidal thoughts resembling a symptom evaluation, accounting for temporal course, frequency and duration, and triggers); we actively engaged novice professionals without clinical training to deepen reflexivity on the intersection of our theoretical and academic background with the analytic data.

The analysis led to an extensive array of initial codes, which were refined and classified into several superordinate categories. Saturation, discussed in relation to the low-level descriptive account of codes, was attained after reviewing roughly 70% of the posts, but we decided to include all posts in this report. To allow room for reflection, we alternated periods of deep immersion with the dataset and moments of distancing from the analysis, which in total spanned over a year. A two-month hiatus was taken between the first arrangement of the descriptive summary and the beginning of the interpretative analysis.

We followed the Consolidated Criteria for Reporting Qualitative Research (COREQ) guidelines (see Supplementary Table 2 for checklist; Tong et al., 2007). We obtained the approval of the local ethics committee. Following similar papers, we slightly rephrased direct quotes without altering their meaning to avoid back-research identification of posts (Mason et al., 2021).

## 3 Results

### 3.1 Descriptive summary

**Figure 1:**
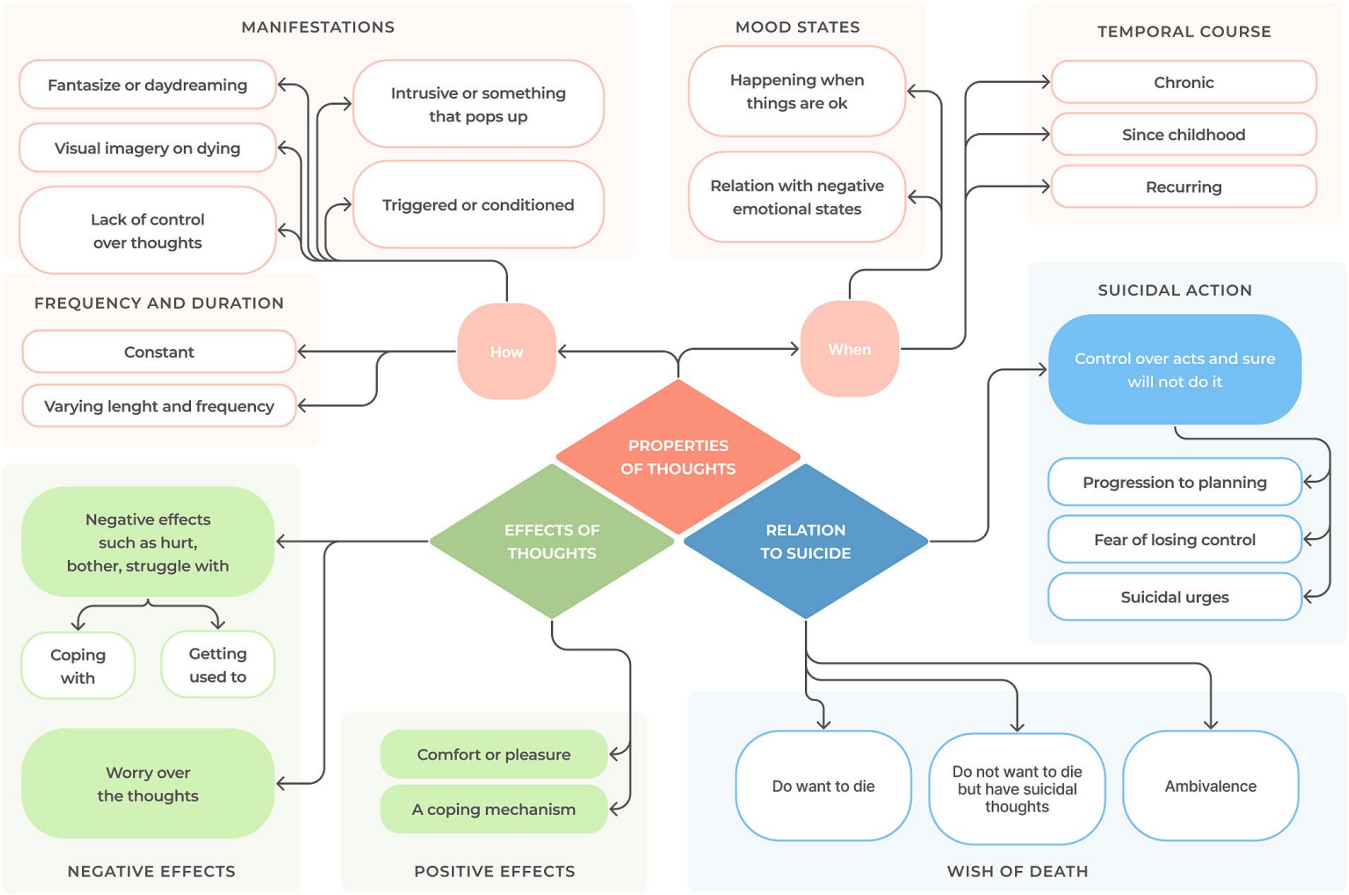
Topic summary of descriptive categories and codes. Notes: While some codes present overlaps, they were not unified due to their intrinsic nuances. “Intrusive or something that pops up” refers to suicidal thoughts appearing unexpectedly in the mind; while this also fits “lack of control over thoughts,” the latter was retained to describe individuals purposefully attempting and failing to suppress thoughts. Similarly, “daydreaming about death” refers to verbal, imagistic, and all forms of fantasizing about dying (usually perceived pleasantly), whereas “visual imagery of dying” specifically refers to a visual picture of death that may be part of a daydream or an intrusive, punctual, and unwanted cognition.

We identified 28 low-level codes directly relevant to the expression of suicidal thoughts, which were grouped under three categories describing the distinct facets of the phenomenon (see Figure 1 for a domain map). Summarizing categories were named **properties of thoughts** (containing two subcategories: “how,” for the form in which thoughts manifest, and “when,” accounting for the associated circumstances and time course), **effects on the person**, and **relation to suicide**. For each category, a codebook with illustrative data extracts is, respectively, presented in Tables 1–3. A more comprehensive array of supporting quotes can be consulted in Supplementary Table 3.

**Table 1:** Codebook and illustrative extracts composing the category “properties of thoughts” (subcategories: “how” and “when”).

Properties of thoughts: how	
**Fantasizing or daydreaming about death** *“I keep imagining my suicide.. I imagine not only the act itself but also the possible reactions of those closest to me. I wonder, who would find me? You see, when I imagine this act, I do it in a way that is least intrusive to those around me. Car accidents are out of the question, overdose is a possibility, however, the idea of my cats not understanding why I’m not moving does not sit well with me. No, when I imagine doing this, I am at my favorite place in this city I love. It seems like a sick irony that I would want my life to end in a place of such beauty. But hey, this is my imagination. So, in reality, the question of who would find me would be broad, and the possibilities would be endless.”*	**Visual imagery on dying** *“I do not know if it’s despair, depression, or a strange new evolution of my daily passive thoughts about how it would be “so simple” to drive off this bridge and “everything would end, and I could rest if maybe I just shoved this awl I’m holding into my larynx.”* *“Is it ideation in case I imagine myself dying and not killing myself? I imagine dying from various violent circumstances several times a day.”*
**The thoughts as intrusive** *“Invasive and nonfunctional suicidal thoughts. I do not have means to kill myself, fortunately and unfortunately. But it continued throughout the day today, constantly: ‘it’s not worth living,’ ‘you have no future, and if you did, it would only bring more pain,’ ‘could you end it by killing yourself?’”*	**Lack of control over thoughts** *“Suicidal thoughts keep running through my head, and I cannot distract myself from them.”* “*I cannot help, it feels like an addiction.”* **Thoughts being triggered or conditioned** *“The slightest feeling of discomfort in my life and then I think about suicide”*
**Constant thoughts** *“These thoughts will not stop. Suicidal ideation is the only thing running on my mind morning, noon and night.”* *“Suicidal ideation became a tinnitus”*	**Thoughts of fluctuating frequency and duration** *“It comes and goes with time and fluctuates in how strong it is but usually at the best it pops very briefly like once a week and at my lowest it at least pops up twice in an hour and lingers on”* *“The thought crosses my mind, I keep it marinating for a minute, and then it’s over.”*
Properties of thoughts: when	
**Chronicity** *“Could I even heal from this chronic suicidal ideation? Going on for about a decade now.”*	**Since childhood** *“I was five or six and I remember I legitimately wanted to die”* **Recurring** *“So suicidal ideation has been coming and going for the last years”*
**Relation with depression and negative emotions** *“Recently some stress made my depression unbearable. I find myself constantly daydreaming about suicide. I cannot think about anything else except how much I want to die.”* *“My thoughts only appear when I’m feeling emotionally exhausted.”*	**Happening when things are ok** *“Is it normal to still have thoughts of suicide while feeling well? Sometimes I could even be having a good day and it’s still there”*

**Table 2:** Codebook and illustrative data extracts composing the category “effects on the individual.”

**Comfort or pleasure** *“Sometimes it’s just weirdly comforting to google how much panadol it would take or other methods, not because I’d actually do it, just because I like knowing it’s there”* *“Those thoughts have a strangely pleasant aspect.”*	**A coping mechanism** *“There are major reasons but sometimes it’s about anything that triggers the feeling. It’s now a coping mechanism. And that gets me stuck in a vicious cycle.”* *“I believe this is a coping mechanism. It’s not healthy, but it gets you to sleep and keeps things going for another day. But it’s so harsh to imagine dying and actually getting comforted by that.”*
**Worry** *“Basically I’m SO nervous about this, it ups my anxiety from 0 to a 100”* *“Are these thoughts worrisome?”*	**Negative effects** *“It’s annoying and it actually hurts”* *“It’s like an addiction, I cannot stop. Either I’m thinking about suicide or I’m thinking about hurting myself. And if I’m not thinking about any of those, I’m not thinking about anything. I’m a shell of a person who never got to live.”*
**Coping with the thoughts** *“Not taking the ideation so seriously has helped me a lot. People panic when they encounter involuntary suicidal thoughts, begin to compulsively block them, but this further attracts their attention and anxiety. Instead, try not to concentrate on them and let them pass. I do not willfully ignore them, but I do not give them my time or attention so they cannot set the music”*	**Getting used to the thoughts** *“I do not know how to combat them, but I it’s good to acknowledge they are passive”* *“I’ve gotten very used to them so I do not worry anymore.”*

**Table 3:** Codebook and illustrative data extracts composing the category “relation to suicide.”

Relation to wishing to die	
**Do not wanna die but have suicidal thoughts** *“I do not want to die either, but my suicide ideation is strong. It’s a belief that I should not be alive, to get the world rid of this broken thing, yet I’ve always rebelled against this idea.”*	**Articulating the thoughts as something dissociated from their actual wish** *“I can say I’m not suicidal, but my mind tries to convince me of something else.”* *“I feel like shit and my brain does not wish to be alive at the moment”*
**Do want to die** *“I have suicidal thoughts because I wanna die, I’ve tried”*	**Ambivalence** *“I keep thinking about death nonstop but a small part of myself still wants to live.”*
Relation to suicidal behavior	
**Control over acts and sure will not do it** *“I am not at risk for suicide. I’m aware of the effects that it would have on those who love me, I do not ever consider it as an option. But the thoughts still happen.”*	**Fear of losing control or buckle to the thoughts** *“I do not know how to deal with the thoughts anymore. I’m afraid I’ll finally gain enough courage at some point”*
**Urges** *“Tonight, while driving home after leaving a friend’s house, I was just screaming and crying the whole way, wanting to veer off the road and crash into a tree.”*	**Progression (to planning or acting)** *“But when depression came again the thoughts turned to passive suicidal ideation and soon after actual suicidal ideation (as googling for methods, searching trying to save up money for a gun, etc)”* *“Usually my thoughts build up to the point shit hits the fan, when I end up spiraling out of control”*

### 3.2 Interpretative synthesis

Three higher-level themes identified core meanings and connections across codes and categories; the invasive and conditioning character of suicidal thoughts was the salient attribute interweaving this interpretative analysis. Each theme is narratively presented alongside supporting quotes.

#### 3.2.1 Imagining escapes: relief and pain in pictures of suicide

Beyond a mere discursive consideration, suicidal thoughts manifest primarily through daydreaming and visual imagery. The imagined suicidal act is experienced initially as a release from pain that eventually evolves into an all-consuming experience. Fantasies revolve around possible methods, circumstances and repercussions, with elaborated considerations such as who would find the body, how people would react, or who would take care of their pets. The mental imagery is vivid and often violent, revolving around incidental, “fortunate” deaths (car accidents and sudden illness), or non-incidental, proactive suicide acts. Those images focus on the very moment of death and the immediate aftermath of a deceased and wounded body: *“I visualize so many scenarios, my brain splattered on the wall, a steel bar running through my head, or violent car accidents.”* Although disturbing, mental images and fantasies function as a means of psychological escape, with the contemplation of death viewed as a coping mechanism that brings a sense of peace - “a *sweet release” -* under adverse situations or feelings: *“I keep imagining falling sick to a disease that will not let me live any longer so I could just stop stressing about things.”*

Yet, this is a deceptive mechanism. While bringing instant comfort, suicidal thoughts concomitantly lead to negative impacts on daily life experiences and may eventually become pervasive. Resembling a conditioning process, the pathways to suicidal thoughts can get amplified to a point that even minor inconveniences can unleash them: “*Why is my automatic response to everything = suicidal ideation. Any stressful event my mind jumps to “kill yourself, things will not get better.”* Experiencing thoughts is a cause of distress, pain, and worry, and may impact activities, impede motivation, and hinder future planning; such negative effects may be overwhelming, and struggling users seek advice on the board on coping strategies to ease the thoughts and their impacts.

**Quotes illustrating fantasizing and visual imagery on suicide and its resulting sense of comfort.**

*“I constantly fantasize about suiciding when under stress. I imagine hanging myself, jumping off a ledge, slitting my wrists. I imagine how others will react. If people will get sad, if I will be remembered, if my parents will get depressed.”*

[**Properties of thoughts**: daydreaming].

*“I find comfort in these thoughts. I enjoy hearing the train pass by; thinking about suicide there. When I go out for a walk with my dogs, I look at every car that passes; I look at the wheels. I find great comfort in imagining diving under the wheels.”*

[**Properties of thoughts:** visual imagery] [**Effects on the person**: comfort].

**Quotes illustrating the conditioning process of suicidal thoughts and their negative consequences**

*“Once you have suicidal thoughts, it becomes an incurable disease. It’s very hard to avoid your mind from going there involuntarily*. *This is slowly killing me, consuming me from inside, leaving only an empty wreck. Some days, it’s all I can think about. It’s as if that’s all that’s left of me, my brain reduced to this black-and-white thought of life or death, and though I may have fleeting glimpses of how irrational it all is, I forget those glimpses as quickly as they appear, and I’m back to the ‘to be or not to be?’ loop.”*

[**Properties of thoughts:** chronic; conditioned; constant] [**Effects on the person:** negative repercussions].

*“This has been happening every day. My first thought when getting out of bed this morning was ‘I should just die’. These thoughts are unpleasant as they demotivate me from doing my tasks throughout the day, making me feel useless, which leads me to contemplate suicide. I hate it because it’s an endless fucking cycle.”*

[**Properties of thoughts:** constant] [**Effects on the person:** negative repercussions].

#### 3.2.2 Integrating thoughts into self: intricate relations with suicidal wish and behavior

People do not describe having suicidal thoughts as a logical deduction of pondering circumstances and feelings, but rather as intrusive experiences invading their consciousness against their will, at times without apparent justification. This is reflected in accounts of suicidal thoughts as an autonomous entity that individuals had no active role in formulating, as in *“I do not wanna die, but my brain insists that I should”* and *“my thoughts continue to drift in that direction.”* This prompts a process of meaning-making of such unexpected thoughts, pondering over what they imply, the person’s wishes to live and die, and possible risks of suicidal action. Some individuals unequivocally state they do not desire death: *“Actually, I only have thoughts about dying or simply disappearing, but I do not want to kill myself.”* Some firmly assert they do have a suicidal wish: *“I do not get the ‘intrusive thoughts’ thing. I do not think about it out of nowhere, I think about it because it is not worth living.”* More often, integrating these thoughts proves challenging, with users engaging in introspective questioning about their true wishes and providing reasons and justifications for each side of the internal conflict: *“My mind is constantly flooded with thoughts like ‘You should kill yourself’ and I am unable to move past it, I give in and listen to those thoughts.”* In such cases, understanding the very expression of thoughts proved a valuable resource for diminishing their relevance and navigating the experience: *“It’s a relief to stop the ‘what is wrong with me’ and go to ‘oh, this silly thing again, that’s alright’.”*

The perception over the risk of incurring into suicidal acts appears to operate in another level, without a linear relation to wishing or not to die. Complete control over their actions is reported even by people wishing to die: *“They are completely passive, I am 100% sure that I will not kill myself.”* On the other hand, people without desires of ending their own lives show concerns about losing control: *“I have always been able to control my impulses, manage my emotions despite feeling depressed. Now, I worry if I am about to lose control.”* There are also suicidal urges, with a sense of not trusting themselves should opportunities appear or experiencing sudden moments of feeling in the edge of suicidal behavior: *“I was driving and could not get rid of the ‘I could just crash my car at 90mph,’ almost felt a physical impulse as my mind stopped my arm from turning the wheel.”* Such perceptions of wish and control were not static, being prone to change over time either in the form of soothing into an enhanced sense of control or progressing from circumscribed thoughts to wishing, intending, planning or acting: *“I’ve been fantasizing about suicide more and more, and I feel that the spiral that leads to actually committing the act is very close by.”*

**Quotes illustrating the invasive character of suicidal thoughts in people who do not wish to die.**

“I feel like my mind is constantly finding a way to justify why suicide is best for me. The sick part is, I can logically look at all that I have written and understand and acknowledge that suicide is not something I \*want.\* [...] However, I keep picturing my suicide. I keep imagining a world where I am no longer a part of it. Where I am not around to make someone smile, to hear my mother’s voice or heartbeat when she speaks; where I do not receive wonderful life advice from my father. All the little things that make life worth living, everything I have worked so hard to have and appreciate, sometimes does not feel strong enough to overcome these thoughts.”

[**Properties of thoughts:** intrusive; constant; daydreaming] [**Effects on the person:** negative repercussions] [**Relation to suicide:** do not want to die but have suicidal thoughts; ambivalence].

*“Today, I’ve been having really strange thoughts that I believe to be suicidal ideation... and it’s honestly freaking me out. I’m fine, but sometimes my mind wanders to imagining cuts on my wrists, which is completely opposite of who I am and anything I’ve ever thought of. I know I would never actually hurt myself, but the thoughts alone are scaring me.”*

[**Properties of thoughts**: intrusive; visual imagery] [**Effects on the person:** worry] [**Relation to suicide**: do not want to die but have suicidal thoughts; control over actions].

**Quotes illustrating the progression of suicidal thoughts and fears related to suicidal behavior**

*“How to tell a counselor that you have suicidal thoughts and they are getting worse?*

*I do not want to act on them, though the thoughts are intense, I do not want to hurt myself, and I do not want to hurt my mother. However, I do not know how much longer I can handle this burden. It’s so constant, so heavy; when I get stressed, my mind goes to suicide, I have difficult thoughts about hurting myself. When I’m scolded at work (my boss is a jerk), I speak cruelly to myself to soften the blow. I’ve had these thoughts before, so strong, but I opened up and survived.”*

[**Properties of thoughts:** triggered; constant] [**Relation to suicide:** do not want to die but have suicidal thoughts; fear of losing control; progression].

*“As long as I can remember I’ve wanted to die. Generally it’s a “wishing to be dead as it’d be better for everyone” or a “i would not move if a car was coming towards me.” But there’s been so many times that I considered killing myself.”*

[**Properties of thoughts:** chronicity; visual imagery; daydreaming] [**Relation to Suicide**: progression].

#### 3.2.3 Stuck into thoughts: long-term nature of suicidal cognition

Suicidal thoughts are often reported as having a chronic character. A first onset could date as early as childhood and persist throughout a lifetime, either remaining constant or recurring after occasional cessations: “*They never stop, very unfortunately. I’m 45 now and have had suicidal thoughts since I was 7 years old.”* Noteworthy, they are primarily associated with periods of vulnerability and negative emotional states such as low mood, stress, and anxiety. However, their enduring and conditioning nature implies that suicidal thoughts also manifest during neutral and positive emotional states and circumstances, a particularly bothersome realization to some users: *“it’s a thought that I constantly have in the background, regardless of good or bad times (I suppose there are just moments/periods when this thought is stronger than ‘usual’).”* Facing a persistent presence, individuals have to adopt attitudes for living with suicidal thoughts. Many struggle with coping with that ever-recurring entity that negatively affects them; yet some express not caring much about these thoughts anymore after having more calmly accommodated to their lived experience: *“They’re always there. They have become part of my life that I’ve come to accept.”*

**Quotes illustrating the persistent nature of suicidal thoughts.**

*“Suicidal thoughts should always be kind of... there? [...] They always just kinda seem to be there? Occasionally, I hit rock bottom of despair and want to commit, but, like today, it was kind of lingering in my mind. [...] Even on good days or neutral days, it’s like that. I do not want to kill myself, but the thoughts still swarm in my brain.”*

[**Properties of thoughts:** chronicity; constant; invasive; relation to negative emotional states; happening when feeling alright] [**Relation to** s**uicide**: do not want to die; progression].

*“However, what changes is that you learn what triggers these thoughts and what is best to alleviate them. You develop coping mechanisms as best as you can, if not for yourself, then for the people in your life who truly love you. I still have suicidal thoughts to this day, and there are times when I feel ready to finally end it all, but I tell myself to bear this heavy burden because this pain does not compare to the pain you go through when you lose someone you love.”*

[**Properties of thoughts:** fluctuating patterns; triggered] [**Effects on the person:** coping with thoughts; getting used] [**Relation to Suicide**: want to die; control over actions and sure will not to this].

## 4 Discussion

Adopting phenomenology as a lens to big qualitative data, this study reports a thematic analysis of several hundred posts on Reddit describing experiences with suicidal thoughts. We conceptualized the expression of thoughts not as a result of reasoned contemplation, but as invasive images and fantasies of death that can alleviate pain and act as a coping mechanism to some. These thoughts may become pervasive and all-consuming, emerging at progressively slighter discomforts and persisting over a lifetime, including periods of relative well-being. When making meaning of uncontrollable thoughts, people reflect on whether they desire to die or not, perceiving varying levels of control over their actions.

Our focus on the very expression of suicidal thoughts differs from recent qualitative research, which usually investigates the reasons for presenting and overcoming suicidality as a broad concept (Peterson and Collings, 2015; Castro-Ramirez et al., 2023; Søndergaard et al., 2023). Nevertheless, extant literature from different methodological backgrounds supports many of our findings. Suicidal intrusions, characterized by vivid imagery and daydreaming, have been extensively documented in quantitative studies and qualitative descriptions (Hales et al., 2011; Ng et al., 2016; Schultebraucks et al., 2020; De Rozario et al., 2021; Lawrence et al., 2023; van Bentum et al., 2023). Previous research has consistently highlighted the comfort and relief associated with thoughts of death, indicating that the contemplation of escape through suicide serves as a coping mechanism to endure suffering, albeit leading to several distressing effects (Lakeman and FitzGerald, 2008; Sturgeon and Morrissette, 2010; Hales et al., 2011; Schlimme, 2013; Crane et al., 2014; Denneson et al., 2020). There is preliminary empirical corroboration for a conditioning process associated with suicidal thoughts (Williams et al., 2005; Antypa et al., 2010), and such notions are also present in the integrated motivational model that proposes the enhancement of a pathway linking stress to suicidal ideation, and in CBT models of suicidality that argue that suicidal cognitions are reinforced once activated, eventually getting overrepresented in the individual’s repertoire (Wenzel et al., 2009; O’Connor and Kirtley, 2018). We suggest that this conditioning process may be linked to the chronic nature of suicidal thoughts, which is another attribute with empirical support (Denneson et al., 2020; Garakani et al., 2022).

Some of our findings shed additional perspectives on prevailing assumptions in suicide frameworks. Ideation-to-action models propose that suicidal thoughts/ideation result from a combination of factors such as thwarted belongingness and perceived burdensomeness, pain and hopelessness, or defeat, humiliation, and entrapment (Joiner, 2005; Klonsky and May, 2015; O’Connor and Kirtley, 2018). In the phenomenological tradition, suicidal contemplation is often confined to the context of despair (Schlimme, 2013). While suicidal thoughts are unequivocally associated with suffering, this hardly accounts for the totality of a phenomenon also expressed by people feeling well and not facing major life adversities. In the existentialist school, the contemplation of death is an arguably by-product of a conscious mind, with recent arguments for expanding our lens to avoid seeing this phenomenon as intricately indicative of mental illness and irrationality (Roberts and Lamont, 2014; Suicidality, 2023). Perhaps a deeper understanding of the various experiences of suicidal thoughts—delineating it as a phenomenon in and by itself rather than studying it under broader terms such as suicidality or suicidal ideation—could enrich and destigmatize suicide research. This is especially relevant for more clearly identifying paths related to suffering and risk.

In this vein, our findings also question the universality of hypotheses proposing connections between suicidal thoughts and behavior. For instance, ideation-to-action theories imply the choice of methods as a stage that is subsequent to suicidal intention (Marie et al., 2020). In our results, we noticed many people claiming no desire or intention to die and still imagining methods of suicide when daydreaming suicidal thoughts. This opens the possibility that planning (or mental rehearsal) could occur before intending or wishing to die, even if not in a purposeful way, reflecting indications that mental imagery increases the risk of behavior (De Rozario et al., 2021; Lawrence et al., 2022). Nonetheless, imagining violent scenarios of suicide also triggered concern in individuals. This may imply a protective function through alarming individuals, compelling them to find ways to avoid the escalation of suicidality. This reflection could potentially favor reasons for living in the internal debates over life and death, including well-described factors such as the fear of suicide (Linehan et al., 1983; Jobes and Mann, 1999). Moreover, our findings suggest a spectrum of suicidal thoughts that may not easily fit into an operationalization on hierarchical stages, as varying combinations of experiences were noticed across the poles “wishing/not wishing to die” and “control over acts/approaching acting.” This has implications for suicidal risk assessment, and we resonate arguments over the need to account for the complex nature of suicidal ideation that could hardly be captured through yes or no questions (House et al., 2020).

We constructed a method around secondary online data to gain insight into the phenomenology of suicidal thoughts across 465 discussions involving unidentified subjects. Big qualitative designs enable access to a massive corpus of experiences not easily captured by traditional methods, potentially optimizing research resources (Fontaine et al., 2020). From a phenomenological standpoint, we argue this type of secondary data offers a unique window into firsthand narratives related to mental health experiences that are less influenced by research conditions. In this sense, our material constitutes naturally occurring data, as we had spontaneous reports of people in the moments they elaborated on their experience (Goldstone and Lupyan, 2016). The anonymity of Reddit further facilitates the reporting of sensitive topics in a well-secured environment that does not require facing a researcher (Sowles et al., 2018; Lea et al., 2020; Belcher et al., 2023).

Online data collection implied several limitations to this analysis. While extensive, our material was mostly constituted by posts of single to few paragraphs, which contained limited context. This is a prevailing challenge for qualitative approaches using big data, and we drew on existing suggestions on how to analyze collections of brief texts (Fletcher and StGeorge, 2011; Robinson, 2022). Nevertheless, we could not tailor questions to specific research purposes, seek clarification of ambiguous sentences, or receive further input from participants on our interpretative claims, and in-depth interviews may lead to richer analytical claims. It is also not possible to describe the sample, including their demographics or mental health conditions. While secondary data collection may include experiences that would be hardly recruited in traditional research, the sample is probably unrepresentative of the general population, being biased toward young, English-speaker participants (Barthel et al., 2016). Online forums have an interactive nature, where discussions evolve as new perspectives and replies are added, potentially enriching research material (Robinson, 2022). While this was evident in our dataset, we analyzed statements separately by user, without considering the temporal course or results of interactions. Lastly, the r/depression forum likely attracts individuals experiencing significant distress, potentially leading to an overrepresentation of this attitude toward suicidality instead of those of other possible users, such as those contemplating existential questions. Additional data pools are needed to broaden perspectives on this phenomenon.

With an innovative big data design for phenomenology, this analysis captured an extensive collection of lived experiences with suicidal thoughts, revealing facets of their expression that extend beyond assumptions underpinning current models of suicidal behavior. These thoughts appear as vivid imagery and daydreams, being described as intrusive by people who struggle to control and make meaning of their occurrence. People who endorse suicidal thoughts may or may not wish to die, as well as perceive control over their actions or fear attempting suicide, with varying and complex combinations across such experiences that do not fit into hierarchical models of suicidality. While bringing comfort during adverse mental states, suicidal thoughts are deemed a deceptive coping mechanism, becoming pervasive and leading to distressing effects. Further explorations, particularly with in-depth interviews, are warranted to delineate the various expressions associated with suicidal thoughts and the pathways linking them to other suicide-related constructs, offering additional inputs for current models of suicidality.

## Data Availability

The original contributions presented in the study are included in the article/Supplementary material, further inquiries can be directed to the corresponding author.
