# Supplementary material for: “I don’t wanna die, but my brain insists that I should”: a big qualitative data approach to the lived experiences of suicidal thoughts
Source: Front Psychol. 2024 Aug 27;15:1420287. doi: 10.3389/fpsyg.2024.1420287 (PMC11385620; doi:10.3389/fpsyg.2024.1420287)
Supplement: Supplementary file 1 [file Data_Sheet_1.PDF]

Supplementary Material

Title: “I don’t wanna die, but my brain insists that I should”: a big qualitative data approach to the lived experience of suicidal thoughts

Supplementary Table 1 - Script developed in Python for extracting **2**

Supplementary Table 2 - Consolidated criteria for reporting qualitative research (COREQ): checklist **3**

Supplementary Table 3 - Codes and illustrating data extracts **8**

Supplementary Table 1 - Script developed in Python for extracting

```
import praw
import pandas as pd
ARQUIVODETEXTO = []
CONTAGEM = 0
reddit = praw.Reddit(client_id="FhwMVsHulwwkzpv25PmDuQ", \
                client_secret='2rsE_yQVfHJkQOp1IUHyn1u8O4RmHg', \
                user_agent='MyBot/0.0.1', \
                username=USERNAME, \
                password=PASSWORD)

TARGET = reddit.subreddit('Depression').search("SEARCH STRING", limit=5000,
params={'include_over_18': 'on'})
for TOPICO in TARGET:
   CONTAGEM = CONTAGEM + 1
   print("!POST NÚMERO!: ")
   print(CONTAGEM)
   print("!TITLE!: " + TOPICO.title)
   print("!POST!: " + TOPICO.selftext)
   COMENTARIOS = TOPICO.comments
   for COMENTARIO in COMENTARIOS:
         try:
            print("!COMMENT!: " + COMENTARIO.body)
            if len(COMENTARIO.replies) != 0:
               for ANSWERS in COMENTARIO.replies:
                  try:
                     print("!ANSWER!: ", ANSWERS.body)
                     if len(ANSWERS.replies) != 0:
                        for SEC in ANSWERS.replies:
                           print("!SECOND LEVEL ANSWER!: ", SEC.body)

                           if len(SEC.replies) != 0:

                              for THIRD in SEC.replies:
                                 print("!THIRD LEVEL ANSWER!: ",THIRD.body)

                                 if len(THIRD.replies) != 0:
                                    for FOURTH in THIRD.replies:
                                       print("!FOURTH LEVEL ANSWER!: ",FOURTH.body)



                  except:
                     print("!ERROR!")
                     pass
         except AttributeError:
```

pass

Supplementary Table 2 - Consolidated criteria for reporting qualitative research (COREQ): checklist

**No. Item**	**Guide questions/description**	**Reported on Page #**
**Domain 1: Research team and reflexivity**		
Personal Characteristics		
1. Inter viewer/facilitator	Which author/s conducted the interview or focus group?	Not applicable
2. Credentials	What were the researcher’s credentials? E.g. PhD, MD	Title page
3. Occupation	What was their occupation at the time of the study?	Title page
4. Gender	Was the researcher male or female?	Page 6
5. Experience and training	What experience or training did the researcher have?	Page 6
Relationship with participants		
6. Relationship established	Was a relationship established prior to study commencement?	Not applicable

7. Participant knowledge of the interviewer	What did the participants know about the researcher? e.g. personal goals, reasons for doing the research	Not applicable
8. Interviewer characteristics	What characteristics were reported about the inter viewer/facilitator? e.g. Bias, assumptions, reasons and interests in the research topic	Not applicable
**Domain 2: study design**		
Theoretical framework		
9. Methodological orientation and Theory	What methodological orientation was stated to underpin the study? e.g. grounded theory, discourse analysis, ethnography, phenomenology, content analysis	Page 5
Participant selection		
10. Sampling	How were participants selected? e.g. purposive, convenience, consecutive, snowball	Page 5
11. Method of approach	How were participants approached? e.g. face-to-face, telephone, mail, email	Page 5
12. Sample size	How many participants were in the study?	Not applicable
13. Non-participation	How many people refused to participate or dropped out? Reasons?	Not applicable

Setting		
14. Setting of data collection	Where was the data collected? e.g. home, clinic, workplace	Page 5
15. Presence of non-participants	Was anyone else present besides the participants and researchers?	Page 5
16. Description of sample	What are the important characteristics of the sample? e.g. demographic data, date	Not applicable. Discussed in Page 19.
Data collection		
17. Interview guide	Were questions, prompts, guides provided by the authors? Was it pilot tested?	Not applicable
18. Repeat interviews	Were repeat interviews carried out? If yes, how many?	Not applicable
19. Audio/visual recording	Did the research use audio or visual recording to collect the data?	Page 5. A textual recording of available data.
20. Field notes	Were field notes made during and/or after the interview or focus group?	Not applicable
21. Duration	What was the duration of the interviews or focus group?	Not applicable
22. Data saturation	Was data saturation discussed?	Page 6

23. Transcripts returned	Were transcripts returned to participants for comment and/or correction?	Not applicable
**Domain 3: analysis and findings**		
Data analysis		
24. Number of data coders	How many data coders coded the data?	Page 5
25. Description of the coding tree	Did authors provide a description of the coding tree?	Page 6
26. Derivation of themes	Were themes identified in advance or derived from the data?	Page 5
27. Software	What software, if applicable, was used to manage the data?	Page 5
28. Participant checking	Did participants provide feedback on the findings?	Not
Reporting		
29. Quotations presented	Were participant quotations presented to illustrate the themes/findings? Was each quotation identified? e.g. participant number	Table 1 to 4, Supplementary Table 2

30. Data and findings consistent	Was there consistency between the data presented and the findings?	Page 6 to 16
31. Clarity of major themes	Were major themes clearly presented in the findings?	Page 6 to 16
32. Clarity of minor themes	Is there a description of diverse cases or discussion of minor themes?	Page 6 to 16

Supplementary Table 3 - Codes and illustrating data extracts

	**Theme: Properties** (Sub Theme: How)
Fluctuating frequency and duration	This happens only once a week or several times a day.<br><br>Suicidal ideation used to be fleeting but is now a daily constant<br><br>I have periods of really intense and sudden depression where I want to kill myself, which can last from a few hours to a few weeks.<br><br>All day, every day, especially at night.<br><br>Furthermore, it is not correct to say every day, but rather several times a day for months. To be exact, 4 months to date.<br><br>Way too often, many times every hour. Other days, just a few times a day.
Constant or non stop	They're always there. They have become part of my identity.<br><br>it does never go away<br><br>Suicidal thoughts should always be kind of... there? [...] They always just kinda seem to be there? Occasionally, I hit rock bottom of despair and want to commit, but, like today, it was kind of lingering in my mind. [...] Even on good days or neutral days, it's like that. I don't want to kill myself, but the thoughts still swarm in my brain<br><br>I have to struggle with almost constant thoughts of suicide.
Lack of control over thoughts	It's like an addiction, I can't stop. Either I'm thinking about suicide or I'm thinking about hurting myself. And if I'm not thinking about any of those, I'm not thinking about anything. I'm a shell of a person who never got to live.<br><br>It's very hard to avoid your mind from going there involuntarily once you start thinking seriously about suicide
Intrusive or something that pops up	I can't make the intrusive suicidal thoughts I've been having go away<br><br>"Invasive and nonfunctional suicidal thoughts. I don't have means to kill myself, fortunately and unfortunately. But it continued throughout the day today, constantly: 'it's not worth living,' 'you have no future, and if you did, it would only bring more pain,' 'could you end it by killing yourself?'"

	The fact that I have a lot going for me like family, friends, a job, doesn't stop the thoughts from being intrusive and trying to kill me.<br><br>Yet my thoughts continue to drift in that direction<br><br>For the past 2 years I have thought about dying or committing suicide every day, but I have no plans to do that and I know I wouldn't. Nevertheless, these intrusive thoughts accompany me on a daily basis and are most intense when I start planning for the future or when I am under pressure. If things go wrong at work? I imagine myself taking pills. If I think about my life in 5 years? A thought about walking in front of a bus<br><br>I keep finding myself thinking about cutting myself or killing myself. It's as casual to think about it as it is to think about dinner. It's been months like this and for some reason I realized today that I'm not okay.<br><br>That's why I can't understand why suicidal thoughts are with me daily now, again. I have them during more neutral activities like cleaning. I have them while watching something. I have them when I'm walking outside and the weather is nice and there's nothing wrong. the only times (but not always - but i'd say 90% of the time) i don't have them is when i'm meeting up with my friends (but it still makes me feel guilty because at the end of the day i have the feeling i've wasted another day by not exercising more, studying more, doing more for my "future self", etc.)<br><br>I started taking 200 mg of Wellbutrin again about 4 days ago. I feel like it's helping me and improving my mood, although it has slightly increased my anxiety. I plan to talk to my doctor about combining Wellbutrin with Lexapro or something similar. Today, I've been having really strange thoughts that I believe to be suicidal ideation... and it's honestly freaking me out. I'm fine, but sometimes my mind wanders to imagining cuts on my wrists, which is completely opposite of who I am and anything I've ever thought of. Should I stop taking it? Is this a normal side effect? I know I would never actually hurt myself, but the thoughts alone are scaring me.<br><br>In the last year, my suicidal ideation has gone from being a very small part of my life to consuming nearly 90% of my time.
Visual imagery	I have vivid and intense visualizations of killing my husband or killing myself.<br><br>That's it, I imagine a car hitting me when I cross the street.<br><br>Sometimes I'm walking to work and a truck is coming down the street, I think yes, there's a possibility I could jump in front of it. Or when I go out for a bike ride on the trails and I'm going to go over the big bridge and I think I could jump off that now and be done with it.<br><br>I just listen to music and visualize someone killing me all day long.<br><br>I don't know if it's despair, depression, or a strange new evolution of my daily passive

	thoughts about how it would be "so simple" to drive off this bridge and "everything would end, and I could rest if maybe I just shoved this awl I'm holding into my larynx."<br><br>When I'm stressed, I fantasize about suicide nonstop. I imagine myself jumping off the ledge, I imagine myself hanging, I imagine myself slitting my wrists. For some reason, however, I think particularly about how others will react. Will they be sad? Will my brother follow in my footsteps? Will my parents be depressed? Will I be remembered?<br><br>I find comfort in these thoughts. I enjoy hearing the train pass by; thinking about suicide there. When I go out for a walk with my dogs, I look at every car that passes; I look at the wheels. I find great comfort in imagining diving under the wheels.<br><br>Accident, sudden illness, suicide, I hear you. That sweet release. Oblivion. I try to remember, when I'm in that place, that whether I go tomorrow or in a few years, either way it's a blink of an eye in the grand scheme of things. That before disappearing forever, I should face life head-on for as long as I can.
Fantasize or daydreaming	I keep imagining falling sick to a disease that won’t let me live any longer so I could just stop stressing about things and worrying<br><br>Anyway, mainly due to a tremendous self-hatred, I think about killing myself every day. However, I have to say that often thinking about my death makes me feel good. I can think about it in different ways. Like maybe there would be one final party with my friends, after which I would kill myself. Or maybe I would kill myself during a drug-fueled drinking binge. Or perhaps I would take one last trip around the city, talk to people, meet new people, after which I would kill myself.<br><br>There are times when I wish I could plan a car accident.<br><br>To be honest, sometimes I really enjoy thinking about suicide. I like to imagine myself dying in different scenarios. It feels calm.<br><br>I just can't help but have fantasies about what it would be like to kill myself, in a pleasant, easy, and painless way, like carbon monoxide poisoning or a heroin overdose. I've had enough pain, and I don't want it to hurt. Just a pleasant and soothing way to go because I'm tired of the cards life has dealt me.<br><br>I've been having the same feeling, imagining that I become ill with a condition that won't allow me to live for long, and then I could stop worrying and stressing about everything. Every now and then, I think to myself: "I doubt I'll live past 25, so maybe it's not even worth bothering about."<br><br>I keep imagining my suicide. This doesn't mean that I will actively kill myself in the near future, as I have managed to go two years without self-harming, thank you very much. However, I can't ignore the simple fact that these thoughts don't subside when I'm alone. I imagine not only the act itself but also the possible reactions of those closest to me."<br>I wonder, who would find me? You see, when I imagine this act, I do it in a way that is least

	intrusive to those around me. Car accidents are out of the question, overdose is a possibility, however, the idea of my cats not understanding why I'm not moving doesn't sit well with me. No, when I imagine doing this, I am at my favorite place in this city I love. It seems like a sick irony that I would want my life to end in a place of such beauty. But hey, this is my imagination. So, in reality, the question of who would find me would be broad, and the possibilities would be endless.<br>Who would tell the kids at my job where I went? I hope they don't disclose this news to them because they already have enough problems. I wouldn't want them to believe that they are not strong or resilient just because a close staff member has disappeared. I would hope they would tell them that I moved.<br>Who would tell my parents? My dramatic, distant, and not-so-close family? For my siblings to realize that their sister wasn't strong enough to truly overcome these dark thoughts, or that I wasn't the miracle my parents had hoped for. To tell my nieces that their aunt won't be coming home again or my nephew that his best friend is gone? I would hope they would embrace each other tightly and remind themselves of how important they are, because my family doesn't do it frequently enough.<br>Who would tell my love? Oh, what a sweet soul. I know how it would affect her, simply because she has already expressed her feelings if I were to suddenly disappear. She would believe it was because of her, and I would hope that those around me would be able to express to her how happy she made me. How I would light up like New York City when her name was mentioned, or how I always wanted to ensure she was well. How almost all the bad things would disappear simply because she entered the room. How, when I looked into the future, I saw her by my side. How it wasn't her fault that I couldn't conquer the monster that lived in my mind long before she even knew who I was.
Triggered or conditioned	The solution in my head, whenever I come across anything, from a small inconvenience to a big problem, is just to kill myself so that it all goes away.<br><br>Someone else sees something, just something small, that triggers you, and your immediate thought is suicide.<br><br>My mind immediately jumps to that when something sad or bad happens to me.<br><br>Once you have suicidal thoughts, it becomes an incurable disease. At least that's how it has been for me. It's slowly killing me, consuming me from inside, leaving only an empty wreck. Some days, it's all I can think about. It's as if that's all that's left of me, my brain reduced to this black-and-white thought of life or death, and though I may have fleeting glimpses of how irrational it all is, I forget those glimpses as quickly as they appear, and I'm back to the 'to be or not to be?' loop<br>When confronted with things I dislike about myself, my response is suicidal ideation. Whenever I sort of realize things that make me uncomfortable or there are aspects of my personality that I should change, my first thought isn't 'I should work on improving how I behave,' but rather 'God, I should just end my life.' I don't know why, and I can only imagine how manipulative this behavior would be if I ever actually told someone about it. I find comfort in the idea of doing something I know I could never actually do, because I'm

	too useless and pathetic to even attempt to be a better person. You shouldn't be faced with something that needs changing within yourself and then resort to self-pity, that's not how things should work. Yet, I still do it."<br><br>There are main, bigger reasons, and sometimes anything can trigger the feelings/thoughts. I believe it has become a coping mechanism. But it keeps me trapped in a cycle.<br><br>I'm not constantly thinking "I should kill myself", it's more like when for example I'm having a bad day at work, there's a subconscious part of me that says "if it gets too bad, you can just run into traffic".<br><br>[21F] I have experienced varying levels of suicidal ideation. At my lowest point, I resorted to self-harm and actively contemplated methods of suicide. However, on normal to good days, suicidal ideation becomes an ineffective background noise (if habitual). During those moments, a small negative event will trigger a 'I want to kill myself' loop in my mind, but I won't feel the urge to actually follow through with it.<br><br>There are times when I forget that there are people who have never considered suicide, while my mind jumps to it when something sad or bad happens to me.<br><br>I happen to have thoughts like this mostly when I feel burdened by obligations: most of the things I need to do for others, at work, or even for myself, I usually find frustrating, boring, annoying, and worthless. This leads me to think that I wouldn't feel any of these things if I weren't here. It's not a very strong thought, but it is persistent. There isn't much I can do about it, other than to keep moving forward and continue or share my frustration with someone I trust.<br><br>My only source of comfort and joy is video games. So, when things go wrong in video games, my suicidal thoughts skyrocket.<br><br>The slightest feeling of discomfort in my life and then I think about suicide<br><br>Am I the only person who has suicidal thoughts while trying to do schoolwork? Trying to focus on schoolwork literally just makes my brain scream incessantly thoughts of self-harm and I hate that.
	**Properties** - **WHEN**
Chronicity	For the past 5 years, I've hated my life and wanted to end it.<br><br>I was diagnosed with depression in 2017, but I have lived with passive suicidal ideation my entire life.<br><br>I have had depression for as long as I can remember. I was around 9 years old when I started feeling what would later be diagnosed as "depressed." I have been constantly struggling with thoughts of suicide.

	So, I'm 30 years old now and I have been dealing with suicidal ideation since childhood.<br>I have had depression and suicidal ideation for a long time<br><br>I have had depression since I was 12 years old (I'm 23). Along with it came suicidal ideation, to say, thinking how good it would be to die.
Recurring	I realize I'm starting to feel suicidal again.<br><br>Suicidal ideation back again on the menu
Since childhood	I just realized I might have suicidal ideation since 14<br><br>Since I was 10 years old, I have been suicidal. I am currently 25 years old.<br><br>Since my childhood I have suicidal thoughts<br><br>Is there anyone else who experienced depression and suicidal ideation at a very young age? Because no one believes me.<br><br>My first suicidal thoughts started around the age of 7-9, as far as I can remember. These thoughts would mainly arise when I had fights with my siblings or parents, and sometimes when I would hurt myself (not severely) or cry for long periods in bed. I always believed that questioning your existence or wishing you were never born was not normal for a child. I know that depression can occur at a very early age, but I don't think that's the case for me.
Relation with negative emotional states	I fantasize about suicide nonstop when under stress.<br><br>My depression tends to worsen significantly in the week leading up to the start of my menstrual cycle. I cry all the time, I can't get out of bed, I have strong suicidal thoughts and the urge to give up. Nothing seems to help, and there's nothing I can do about it. All I can do is wait because, on the day my period starts, suddenly all the pain almost disappears. It's as if someone just pressed a button, and I become a completely different person. Obviously, I still suffer from depression, but it's at a whole different level.<br><br>Depression is a mother fucker, especially when you have major depressive disorder. It's miserable. I go straight from feeling indifferent to wanting to no longer exist. Being medicated helps, however, there are times when it's very difficult to deal with it. There's no logical reason behind it.<br><br>I only have thoughts about dying or simply disappearing, but I don't actually want to kill myself. Even though I'm still okay having a job and living with my girlfriend in our own house, I still have to cope with and live with depression every day. Most of the time, it feels manageable, but lately, it has been much worse when I think about dying or just not being here anymore because I feel so tired of life.

	I have been suffering from severe depression for years, and due to my attempts, I have been admitted to psychiatric units several times. More stress has been added recently, which is making my depression unbearable. I often find myself daydreaming about killing myself. I really can't think of anything else besides how much I want to die. Moreover, I have tried a variety of medications, but I have treatment-resistant depression as none of them have been effective. I have seen numerous therapists who haven't helped, but they were really terrible therapists, so maybe I should look for a better one? It even happened that one of my therapists opened a CBT book and read out one of the pages to me.<br><br>How to deal with deep depression/suicidal thoughts during the night? I don't believe I would kill myself. I love and care for my family more than anything, but it becomes distressing. I don't let the thoughts bother me, but during the night, when I'm alone, the depression truly intensifies and affects me. I always need to keep myself occupied, or I feel extremely depressed.<br><br>I display warning signs of suicidal ideation when I go through difficult periods.<br><br>I have suicidal thoughts linked to pre menstrual periods. My depression spirals out of control whenever it's close to that time of the month. As I become increasingly moody, I genuinely start thinking about suicide and ending it all.<br><br>I am a 20-year-old woman living in the United States. I am currently living at home with my parents and siblings. My mother is a textbook borderline narcissist who has abused me throughout my entire life. Nobody in my family understands me and they blame me for everything that happens. I am trying my hardest to leave, but I can't because no matter what job I get, it doesn't pay enough for me to live anywhere else besides here, with these people. I don't earn enough money to cover basic expenses, and as a result, my credit is bad. Therefore, I can't qualify for any loans. I don't own a car, so I don't have anything as collateral. I can't afford to go to college, and I have no savings. I can't afford a therapist or financial advisor. I am trapped. The stress is killing me. In addition to all of this, I live with chronic pain, which makes my existence even harder. I don't want to kill myself, but it feels like the only thing I can afford.
Happening when things are ok	Suicidal ideation even when my life isn't that bad<br><br>Today was good. The best I've had this week, in fact. But I am still suicidal.<br><br>Even when having a good day, there's still this thought that i wanna off myself.<br><br>It's not only when I'm feeling really depressed, it comes up all the time.<br><br>I'm not really depressed, but I think I used to be. I tried looking this up but no answers. What's it called when you're not actively looking or making plans to kill yourself, but you wouldn't mind dying and you're not even sad about it anymore? Is this passive suicidal ideation?<br><br>Even when i'm having a good day I still have passive suicidal thoughts. just so weird.

	Suicidal ideation but not depressed? I wanted to ask, even though I know it has been answered a million times, but I can't find an answer that fits my specific situation. I'm not sure if I should post this on /suicidewatch because I'm thinking about it while sitting at work, and I won't go home tonight or in the future to attempt suicide, but I will move it there if people think it's more appropriate.<br><br>I think about suicide a lot. At least once a day, but probably more, and I fixate on it. It's not just a fleeting thought. Recently, the lead singer of my favorite band committed suicide, and when I found out the method, the first thing that came to my mind was, 'Yeah, that's how I would do it too.' Yesterday, while driving down the road, I couldn't shake the idea of, 'Yeah, I could totally just slam my car into this highway median at 90mph,' and I almost felt a physical tension as my conscious mind restrained my arm from turning the wheel.<br>That being said, I don't want to die, so I'm not actively suicidal. Although I was diagnosed with depression ten years ago when I was a Peace Corps volunteer, that diagnosis was largely considered a medical error and, at best, situational.<br><br>I don't mind being alive, I do things that interest me, have fun with the people I love, have an amazing family who loves me, among other things. I feel happy most of the time. I just get really tired sometimes (often), and to some extent, everything feels like a hassle. In areas of my professional life, especially, I feel like I'm always settling for mediocrity and that I'll always be mediocre, kind of lost in what I'm doing (and if someone thinks otherwise and that I'm doing really well, it's because I somehow fooled them). I can't concentrate on ANYTHING. It takes a lot of motivation for me to leave the house and engage in social activities, but when I do, I have a good time, and I travel a lot. Life feels like it's simultaneously flying by and dragging on."
	**Effects**
Comfort or pleasure	Suicidal Ideation feels comforting. I feel safer thinking about ending it than I do confronting the issues that have brought me to an emotional rock bottom. I don't know how to explain.<br><br>I find comfort in fantasies about my own suicide. It brings me so much pleasure. I lie in bed and imagine how shocked and sad people would be. It's amazing to receive all that attention. I wish I could do it to prove how much I'm struggling, so they would finally understand. I imagine what I would write in the suicide note and how each of them would receive the news. Who I would apologize to and who I would blame. It feels like I would receive so much justice. These thoughts bring me comfort. When I walk my dogs, I look at the wheels of every passing car. I find so much comfort in imagining diving under the wheels. I enjoy hearing the train pass by; thinking about suicide there.
A coping mechanism	Suicidal thoughts are comforting. Every time I'm stressed about something, I think to myself, "Well, I shouldn't worry about anything because I'll be dead in a few days anyway."<br><br>I find relief in suicidal thoughts. I just wish I wasn't here anymore. I wouldn't have any more problems if I were gone. A few nights ago, I had a chest pain that I thought was my heart. I

	was anxious until I realized that I could simply die if I went to sleep. Instantly, I smiled and fell asleep. I didn't die. Kind of wishing that had happened.
Coping with the thoughts	What helps me? I have to listen to loud music to distract myself from thoughts when I'm outside, and at night or early evening, I smoke weed to self-medicate, which sort of blocks out the thoughts, although it can intensify dissociation as well. I'm still waiting to start taking some miracle psychiatric medication, but I don't know...<br><br>It's a kind of relief when it goes from "WTF is wrong with me" to "oh, this stupid shit again. Okay."<br><br>I just accepted that they were a part of my existence. I really don't know how to fight them, although it's good that you recognize that they are passive<br><br>At least initially, I believe that what helped me was not taking suicidal thoughts seriously. Many people panic when they have involuntary and intrusive thoughts about suicide. Compulsively, we start blocking them, which only attracts more attention and anxiety. That's not a good strategy. Try to let them pass. If you have practiced meditation before, and if not, I strongly recommend you give it a try. You learn how not to focus on thoughts and let them go. That's how I approach intrusive negative thoughts - I don't give them the power to set the music, I don't intentionally ignore them, I simply don't dedicate my attention and time to them.<br><br>I've tried to diminish the power of this habitual thinking by using humor. With friends I trust, I make jokes about how hilariously minor inconveniences make me want to "die" and how "sad" I am. I don't engage in this behavior around friends who are sensitive to mentions of suicide, and I always try to speak in a way that suggests I'm not being serious. When my friends ask if I'm alright, I explain that my brain still has fixed suicidal ideation as a coping mechanism, and using jokes helps me diminish the power of those thoughts.<br><br>They never stop, very unfortunately. I'm 45 now and have had suicidal thoughts since I was 7 years old. However, what changes is that you learn what triggers these thoughts and what is best to alleviate them. You develop coping mechanisms as best as you can, if not for yourself, then for the people in your life who truly love you. I still have suicidal thoughts to this day, and there are times when I feel ready to finally end it all, but I tell myself to bear this heavy burden because this pain doesn't compare to the pain you go through when you lose someone you love. I know this because my brother took his own life, so all you can really do is continue to give your best.<br><br>I understand that suicide will always remain in the background of my mind, but it is not the destination or a safety net I have always felt that if I failed miserably or embarrassed myself, and if things didn't improve, I could just end it all. However, I have come to accept that my mind will always gravitate towards suicide because of the reason mentioned above. Now is the time to find strategies or safety nets in something different.
Worry	I can get through it even though it can be pretty scary

	The thought lasted maybe 60 seconds and then I snapped out of it and freaked out<br><br>I'm scared because I had the first real suicidal thought in years
Negative effects	It's like everything is consuming my existence, but I don't actually want to kill myself.<br><br>I have already experienced the severe stress to which these thoughts can lead me. I cannot simply 'wish I wasn't here' in a casual way.<br><br>Can’t sleep because of the suicidal ideation, I feel like a total burden existing<br><br>I can't motivate myself because of it<br><br>Suicidal ideation is a difficult thing Eventually it becomes obsessive and all consuming.<br><br>I believe that recently I have been doing okay. Not extremely happy, but not completely depressed either. However, I have been experiencing passive suicidal thoughts for about a week now. In case you don't know what that means, it's when you think about suicide but don't have any actual plan or real motive to do it. This has been happening every day. My first thought when getting out of bed this morning was 'I should just die'. These thoughts are unpleasant as they demotivate me from doing my tasks throughout the day, making me feel useless, which leads me to contemplate suicide. I hate it because it's an endless fucking cycle.
Getting used to the thoughts	That's very relatable. I'm 99.9999% sure that I would never do anything to end it, but it has become a normal part of my day to think about killing myself because my thoughts are very intrusive. Like you, there's always an excuse not to do it, but regardless, dealing with these thoughts is terrible.<br><br>I don't know. I feel like thinking about suicide is now part of my routine. There are moments when I wonder what goes on in the minds of those who don't want to die.<br><br>My subconscious has kind of normalized suicidal thoughts.
	**Relation with suicide**
Do not wanna die but have suicidal thoughts	As much as I don't want to die, my suicidal ideation is quite intense at times. In my mind, there's a belief that I shouldn't be alive and that I should rid the world of this broken thing. However, I have always rebelled against it.<br><br>Does anyone feel comforted by suicidal ideation? I'm not going to kill myself. Firstly, I have many loved ones who would be devastated. Secondly, I don't really want to die.<br><br>Actually, I only have thoughts about dying or simply disappearing, but I don't want to kill myself. Even though I'm still okay having a job and living with my girlfriend in our own house, I still have to cope with and live with depression every day. Most of the time, it feels

	manageable, but lately, it has been much worse when I think about dying or just not being here anymore because I feel so tired of life. I imagine myself disappearing and how that would also be beneficial, as if I were gone, my father's estate wouldn't be divided among so many remaining relatives, and they could benefit from it more than I would. Our family isn't wealthy either, but I figure there's no point in me being here since I wouldn't do anything incredible or great with it either. It really seems like I would be better off completely disappearing, and I have no idea why this thought seems rational at this time. I know it's probably my depression trying to say something that throws me off balance, but it's a thought that I constantly have in the background, regardless of good or bad times (I suppose there are just moments/periods when this thought is stronger than 'usual')."<br><br>I feel like my mind is constantly finding a way to justify why suicide is best for me. The sick part is, I can logically look at all that I have written and understand and acknowledge that suicide is not something I *want.* [...] However, I keep picturing my suicide. I keep imagining a world where I am no longer a part of it. Where I am not around to make someone smile, to hear my mother's voice or heartbeat when she speaks; where I don't receive wonderful life advice from my father. All the little things that make life worth living, everything I have worked so hard to have and appreciate, sometimes doesn't feel strong enough to overcome these thoughts.
Do want to die	I just listened to music and visualized someone killing me all day long. Honestly, I just want to die. I didn't ask to be here. I'm not looking for any advice, just wondering if anyone has had a similar day.<br><br>I want to die. However, at this moment, after thinking about actually killing myself, I'm not going to do it.<br><br>I never have a plan. In 9 years, I've attempted 4 or 5 times. It's a literal decision every day, and that's why it's more than just ideation and not just casual thoughts. I don't even have to be upset. I tried to explain to a therapist that it's more than just being used to thinking about it or having intrusive thoughts, it's actually wanting to die all the time and having no will to live or protect my life. Even though I avoid pain, if I knew something would kill me, I wouldn't stop it because I don't care enough to do so (like being on a railroad bridge and a train approaching, I wouldn't jump off and risk pain to save myself because I would prefer to die).<br><br>I have them because I want to die, I've made attempts before, but my friend found me and stopped me, so I wasn't successful. Usually, my thoughts build up gradually until everything falls apart, and that's when I end up losing control and spiraling.
More than intrusive thoughts	Intrusive thoughts is a term commonly used when referring to individuals with obsessive compulsive disorder who experience unwanted and obsessive thoughts that usually have nothing to do with them as individuals. Someone crossing a bridge, with an absolute will to live, and their mind screaming "Jump! Jump! Do it!" to the point of deeply disturbing them. Obviously, this is not what we experience.”

Ambivalence	There's still a part of me that wants to live, even though I keep constantly thinking about death. I didn't get any help from trying therapy, and even if I wanted to go back, I wouldn't have the financial means to afford it. Besides, even if I continue, I'll have to deal with being in a body that I hate (I'm trans). I'm simply stuck.<br><br>I don't know why, but I thought about killing myself last night. I just sat in my bathtub and in my room crying. I don't want to, but it feels like something I should do. I don't know the reason.<br><br>I have been suffering from depression for a long time. I was first diagnosed with clinical depression when I was 13 until around 15. I had a failed suicide attempt at 14, which resulted in hospitalization. At 18, after giving birth, I experienced postpartum depression for about a year. Apart from minor depressive episodes, I wasn't formally diagnosed again until March. I am now 25 years old.<br>I'm terrified by my first suicidal thought in years. I'm having a bad day. Nothing major, just a damn bad day. But I'm lying here, and suddenly the thought of overdosing on my antidepressants came to my mind, and the worst part is how good it sounds. Like it would be the solution to just end it all. I don't want to die, but I don't want to live either.<br>I have two beautiful children, a marriage with my best friend in the world, and an amazing life together. I don't want to die, but I also don't want to live. I'm very afraid of myself. Does anyone have any advice?
Progression (to planning or acting)	*Suicidal thoughts... turning to plans.*<br><br>*Last week I was in the hospital because my chronic suicidal ideation became unbearable and I started making plans*<br><br>'m not sure if I actually have depression, I think I have some symptoms, but as I said, I'm still in the process of figuring it out. Well, at least I should go to a psychiatrist, but I haven't set a date yet. I'm not really excited about going, but well, what can I do. Basically, I wanted to ask if this is how we feel when we idealize suicide. Edit: I forgot to mention that I've basically started planning my suicide, I just haven't set a date yet.<br><br>My suicidal ideation has shifted from passive to active due to the general election. Faced with the decline of our country, I have started planning exit strategies. I can't see a light at the end of the tunnel. I'm fixated on it, I know, but I have reached a point where I can't stop.<br><br>What should I do? The pressure from university and my addictions have culminated in intense suicidal ideation. Post: I've been smoking about a gram a day using gravity bongs and I've become addicted to the feeling of "ease" I get when I accomplish absolutely nothing. I've been going to bed at 8 am and waking up at 4 pm or even later, becoming nocturnal. I would stay up all night watching nonsense, pornography, anything to pass the time, and wake up feeling depressed, exhausted, apathetic, and I've become really antisocial and hard to talk to. My passive suicidal ideation has become more and more

active, making university really, really hard for me. I've passed all my subjects this year, but I have one last subject to graduate, and I desperately feel like I need that graduation as a win. So much so that I might kill myself if I fail another subject. I realized how serious this is and quit weed cold turkey a week ago, and the withdrawal is truly fucked up. It made everything even worse, and now I'm even more intent on suicide if I fail. I don't know what to do to feel happier anymore. I'm always stressed about university, and I don't see the value in life anymore. What should I do?

suicidal thoughts getting realer and realer
Please do not read this if you're under 18. Hi, I'm a 19-year-old addict, and I've been depressed for as long as I can remember. I have occasional suicidal thoughts, but nothing severe. I've been in and out of psychiatric hospitals because, luckily, I have loving parents whom I'm very grateful for. However, my mother has an autoimmune disease called lupus, which has turned me into a hypochondriac. Sometimes I feel like I'm rotting from the inside out, I can't breathe, everything goes numb, and I can feel my heart beating faster than ever. Recently, I've either been staying locked in my disgusting room for days, drinking myself to death, or I've been popping Xanax again. I was sober for almost a year, but I relapsed a week ago. When I'm not just dwelling in my filthy room, I'm out and I won't come home for days because I'm too busy putting any substances I can find into my body. I can't stay sober for more than a day, and I can barely eat. Now, suicide is a viable option, I have a plan: I'm making an EP called suicide note, with 6 songs, I'm going to release it and then finish everything. I don't want to do this, but I feel it's necessary.
I feel like this is the end as I'm experiencing my suicidal thoughts turning into more than just thoughts. Post: Just now, I felt the strongest desire to die that I've ever felt in my life and started crying uncontrollably. I don't think I've ever felt this suicidal before. In addition to the suicidal thoughts, I've now started planning and looking around me for something. I could barely move due to intense depression and sadness. I don't know what to do. I don't have someone to talk to. I have no family, no friends, no one. I'm going to the doctor tomorrow, but I'm giving myself two options if they can't help me: try to kill myself or use drugs to make me feel somewhat better. I see no future. I have no friends, no education, and my family simply doesn't care as they just mock me. I have no experience, no ambitions because I know I won't achieve anything, and I also know I'll lose interest in the next two damn weeks anyway. I only see darkness. The tunnel is closed on both sides. I'm at my lowest point ever. There's nothing for me. Back in 2015/2016, I was extremely suicidal and I planned how to proceed and started writing the letter. I have no idea why I didn't go through with it. It's almost like I regret it to this day. I would have ended my suffering long ago, but no. Unfortunately, I'm still here.

For years, I have struggled with depression and suicidal thoughts. I haven't told many people about it. And I'm afraid of talking to people about it because I feel like I'm a burden on them. Currently, my parents are spending thousands of dollars on my education and I'm failing in college. From a financial standpoint, they would be better off without me.
Yes, there are ways to get better through therapy, hard work, and talking it out. But death just seems like it would be quick and easy. Living is not enticing to me anymore. The effort of existence is too high. I just want to die. I have learned that suicide is not my only option.

	And I don't think like that anymore. However, at this point, I want to choose suicide over my other options just because it's the easy way out.<br>I've been fantasizing about suicide more and more, and I feel that the spiral that leads to actually committing the act is very close by. Honestly, I don't know why I'm writing this, but maybe it's just to get my feelings off my chest.<br><br><br>How to tell a counselor that you have suicidal thoughts and they're getting worse?<br>I don't want to act on them, though the thoughts are intense, I don't want to hurt myself, and I don't want to hurt my mother. However, I don't know how much longer I can handle this burden. It's so constant, so heavy; when I get stressed, my mind goes to suicide, I have difficult thoughts about hurting myself. When I'm scolded at work (my boss is a jerk), I speak cruelly to myself to soften the blow. I've had these thoughts before, so strong, but I opened up and survived.<br><br><br>When we are depressed, is this how we feel suicidal ideation? First of all, I haven't been diagnosed with depression. My therapist seems concerned about me and said I should see a psychiatrist. Anyway, mainly due to a tremendous self-hatred, I think about killing myself every day. However, I have to say that often thinking about my death makes me feel good. I can think about it in different ways. Like maybe there would be one final party with my friends, after which I would kill myself. Or maybe I would kill myself during a drug-fueled drinking binge. Or perhaps I would take one last trip around the city, talk to people, meet new people, after which I would kill myself. Basically, I wanted to ask if this is how we feel when we idealize suicide. Edit: I forgot to mention that I've basically started planning my suicide, I just haven't set a date yet.
Fear of losing control or buckle to the thoughts	What's really scaring me are these suicidal thoughts. They're constantly being forced into my mind, and I don't disagree with them, I think about it, how and where I could do it. I haven't been allowing myself to go near any high places as I worry I might jump on impulse.<br><br>The fact that things may not improve and that the thoughts may become less passive concerns me.<br><br>I'm terrified because I believe my suicidal ideation is becoming increasingly dangerous every day.<br><br>Why do I feel like therapy is designed to worsen people's situations and bring them to their worst possible mental state? Without going into too much detail, I can't stand my in-person job anymore, and I don't feel qualified for any of the remote positions I'm applying for. The poverty wages offered are not enough to support myself, and I don't feel smart enough or have enough time to learn skills that would qualify me for higher-paying jobs.<br>This makes me feel worthless and makes me think it would be better to spend the little money I have now, buy a gun, and end my life.

	Of course, I would love not to feel this way or have these thoughts, but how can I seek help for these intrusive thoughts when disclosing them will condemn me to an even worse situation?<br>I can't go on like this much longer. My stress, anxiety, and depression have such a strong grip on my perspective that I don't know if I'll ever be mentally healthy enough to escape these thoughts.<br><br>I'm not actively planning but I'm scared it'll get there. I've been struggling for a while but it's getting bad.<br><br>After several weeks of struggling with serious suicidal thoughts, I finally gave in and asked my husband, whom I've been married to for a year, to hide things that I don't trust myself with.<br>The look of fear and pain in his eyes was heartbreaking. He had never seen me suffer so much. It has been almost a decade since I reached this point, long before we met. Even back then, I never openly asked for help when I should have. I promised myself I would never reach this point again, especially now for his sake, but the overwhelming feeling of wanting the pain to stop is sometimes unbearable.<br>But I did it. As hard as it was, I spoke up. I gathered everything I didn't want easily accessible and asked him to put it all away where I wouldn't find it. I promised him that if I needed anything, if I got scared, or if I needed more support than he could provide just by being there, I would call for help. It was scary, shameful, even embarrassing to admit how I was feeling. It hurt even more knowing that he had never dealt with anything like this, and he's probably more terrified than he'll admit. I know I can't take that fear away from him now that it's there, but I also know that this is better than suffering silently and hoping for the best.<br><br>So, while listening to music a few nights ago, a thought crossed my mind. "This would be a good song to die to." Then, I thought about how I would play the song and cut my wrists, letting myself fade away. And it made me feel? Like I could do it if I wanted to? I'm not sure how to describe it. I don't want to die. But are these feelings I have worrisome?
Control over acts and sure will not do it	I've had intrusive thoughts about suicide happening during all my life, but no real urge to act, nor do I want to die or think it'd be better<br>What I told my therapist is that even though I can't control my thoughts, I can control my actions. I have suicidal thoughts, but I am NOT suicidal.<br><br>I would go to a hospital if I truly believed I was in danger because I don't want to die, but I know I'm not at risk. It's just that my mind doesn't want to be alive right now, and I feel terrible.

	Honestly, I never really considered myself capable of doing anything, given the way I react and handle physical pain. I guess that's why I never worried too much about it; I just assumed it was due to the rest of my mental state. I never thought it could change and worsen.<br><br>Just to clarify, I am not currently at risk of suicide. What prevents me from considering it as an option is my understanding of the effects it would have on those who love me. However, the thoughts still persist.<br>Just as a disclaimer: I will never kill myself, I'm not going to do it, I know that to be true.
Urges	I've been dealing with a lot of suicidal ideation lately. I really wanted to do this yesterday. It was so tempting. But instead, I kind of kept myself "paralyzed" where I was, so I couldn't do anything.<br><br>Even though I don't visualize it, I often see opportunities. For example, when I walk on a bridge, I think, "I should just jump off." Or when I see pill boxes in my drawer, I think, "Why don't I overdose by taking all of them at once?"<br><br>After these encounters, I find myself driving home, in the car, just screaming and debating whether I should simply accelerate and crash into a fucking tree at 100 miles per hour.<br><br>Tonight, while driving home after leaving a friend's house, I was just screaming and crying the whole way, wanting to veer off the road and crash into a tree.<br><br>In recent weeks, my suicidal thoughts have been at an extremely high level. At the same time, I'm scared, but I'm really tempted to grab a knife and do something, feel something. I know I shouldn't, I know I really shouldn't even consider hurting myself as a solution, but damn, I hate myself so much and I also think, what's the point of hurting myself? What's wrong with wanting to feel something physical too if I'm already broken inside? Someone please help me think differently... I'm scared of myself and what I could do to myself.<br><br>Suicide is something that I think a lot about, at least once a day (but probably more). I dwell on it, it isn't just a fleeting thought. My favorite band lost its lead singer to suicide recently and when I found out the method, the first thing I thought was "that's how I'd do it too." I was driving down the road yesterday and I couldn't get rid of the idea of "yeah, I could totally just slam my car into this highway median going 90mp" and I almost could feel a physical tension at the same time that my conscious mind restrained my arm from turning the wheel. Having said that, I'm not actively suicidal cause I don't want to die, and although I was diagnosed with depression ten years ago when I was a Peace Corps volunteer, it was mainly considered a malpractice diagnosis and was, at best, situational.
